# Pulmonary Endothelial Extracellular Vesicles Preferentially Interact and Are Processed in Pulmonary Endothelial Cells

**DOI:** 10.1096/fba.2025-00327

**Published:** 2026-04-02

**Authors:** Aritra Bhadra, April Scruggs, April Haven, Morgan Malone, Natalie Bauer

**Affiliations:** ^1^ Department of Pharmacology, Center for Lung Biology Frederick P. Whiddon College of Medicine, University of South Alabama Mobile Alabama USA

**Keywords:** endocytosis, exosome, extracellular vesicle, lysosome, pulmonary, trans‐Golgi

## Abstract

Extracellular vesicles (EVs) regulate vascular injury and homeostasis; however, the intracellular mechanisms for the EV effects are unknown. While EV components and functions are well studied, their cellular uptake and intracellular processing remain unclear. Following our previous work on EVs, we investigated EV uptake mechanisms and organelle localization. Pulmonary endothelial–derived EVs showed preferential interaction with pulmonary vessels, particularly injured vessels. Co‐culture experiments confirmed endothelial cells as exclusive EV targets via energy‐dependent, clathrin‐mediated endocytosis. Intracellularly, EVs colocalized with lysosomes and trans‐Golgi, suggesting degradation and protein recycling pathways. These findings provide insights into temporal EV uptake dynamics and organelle interactions, establishing a foundation for understanding EV processing and content distribution mechanisms.

## Graphical abstract

This graphical abstract illustrates that extracellular vesicles (EVs) are preferentially taken up by pulmonary artery endothelial cells in pulmonary arterial hypertension (PAH). Uptake is enhanced in diseased vessels and occurs exclusively in endothelium, not smooth muscle. EV entry is clathrin dependent and leads to trafficking through endosomes, lysosomes, and the trans‐Golgi network. These findings highlight the selective targeting and intracellular processing of EVs in PAH, informing potential therapeutic strategies.

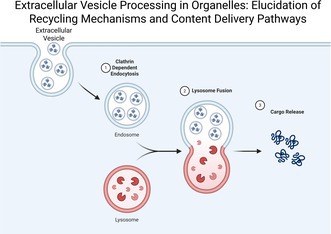

## 1 Introduction

Extracellular vesicles (EVs) facilitate intercellular cargo transfer through membrane‐bound structures released from donor cells [1, 2, 3]. Despite advances in characterizing EV biogenesis and composition, the cellular specificity of EV uptake and subsequent intracellular fate remain undefined. Vascular beds exhibit distinct endothelial phenotypes, yet whether tissue‐specific EVs demonstrate preferential homing patterns is unknown. Furthermore, whether EVs undergo membrane fusion or intact internalization, and how EVs traffic internalized to specific organelles for cargo delivery remain unresolved. We hypothesized that pulmonary endothelium‐derived EVs preferentially accumulate in pulmonary vasculature through endothelial‐specific uptake and undergo clathrin‐mediated endocytosis with directed trafficking to early endosomes, trans‐Golgi, and lysosomes for cargo processing.

## 2 Methods

### 2.1 Cell Culture and EV Isolation

Male Sprague–Dawley rat pulmonary microvascular endothelial cells (PMVECs), pulmonary endothelial cells (PECs), and pulmonary smooth muscle cells (PASMCs) were obtained from the Center for Lung Biology, sorted, characterized via lectin staining, and cultured as previously described [4]. EVs were collected, centrifuged at 1000 × *g* for 10 min, ultracentrifuged at 100,000 × *g* for 1 h at 4°C, resuspended in sterile PBS, and maintained at 37°C [5, 6]. Isolated EVs were labeled with PKH26 red (#MIDI26‐1KT, Sigma Aldrich) or PKH67 green lipophilic dyes (PKH67GL‐1KT, Sigma Aldrich) following manufacturer protocols. Co‐cultures utilized 10‐μm thick polycarbonate transwell inserts (0.4‐μm pores, 4.2 cm^2^ surface area, EMD Millipore Corp., Billerca, MA) pre‐soaked for 4 h. PASMCs (1 × 10^5^) were seeded on six‐well plate bottoms and incubated overnight (37°C, 21% O_2_, 5% CO_2_). After 24 h, PECs (1 × 10^5^) were seeded on transwell upper surfaces.

### 2.2 Sugen/Hypoxia Animal Model for Injury Studies

All experiments were performed with the approval of the University of South Alabama Institutional Animal Care and Use Committee. Male Sprague–Dawley rats (body weight, 240–280 g) were age‐matched and distributed in two groups: Sugen/hypoxia rats and control rats. Briefly, the 8‐week SU/Hx Sprague–Dawley rat model was generated as published previously [7]. Briefly, the extra‐lobar first branches of the pulmonary artery, the aortic arch, the mesenteric artery (approximately 1 mm diameter), and the post‐ to mid‐cerebral artery were isolated from Su/Hx rats following approved termination [8]. To determine whether EVs could interact with vessels in vivo and whether the uptake was altered in pulmonary arterial hypertension (PAH), we used isolated and labeled EVs, with the fluorescent membrane dye PKH26, and injected 50 μg of labeled EVs into the tail vein for 30 min of circulation in control and Su/Hx rats.

### 2.3 Organelle Labeling

Organelle‐specific antibodies and fluorescent labels were optimized to study EV internalization and subcellular localization in PMVECs (Table 1). For fixed‐cell analysis, labeled EVs were incubated with PMVECs (70% confluence, gelatin‐coated 35 mm glass‐bottom dishes) for 0.75–6 h at 37°C, then fixed (2% formalin, 10 min), permeabilized (0.1% Triton X‐100, 10 min), blocked (5% BSA, 1 h), and immunostained overnight at 4°C. Signal detection used VectaFluor Excel amplifier antibodies (15 min, room temperature) and secondary antibodies (30 min); nuclei were counterstained with DAPI in mounting medium. For live‐cell imaging, PMVECs at 70% confluence were pre‐labeled with organelle‐specific dyes (Table 1) before adding labeled EVs (0.75–16 h, 37°C); Hoechst 33342 (20 μg/mL, 20 min) visualized nuclei. Imaging was performed with a Nikon A1R confocal microscope. CellProfiler software identified and analyzed colocalization of EVs with organelles.

**TABLE 1:** Organelle‐specific antibodies and fluorescent labels for cell staining.

Organelle/cell component	Label	Dilution/staining notes	Vendor information
Early endosome	EEA1	1:100 Rabbit polyclonal	Thermo/Pierce PA5‐17228
Lysosome	CellLight Lysosome Transfection	48 h transfection Green fluorescence	Thermo C10507
Cis‐Golgi	GRASP65	1:500	Thermo/Pierce
Network		Rabbit polyclonal	PA3‐910
Trans‐Golgi	TGN38	1:500	Thermo/Pierce
Network		Mouse monoclonal	MA3‐063
Mitochondria	MitoTracker	Red fluorescence	Thermo M22425
Nuclei	DAPI	Blue fluorescence	Vector H‐1200
Nuclei	Hoechst 33342	Blue fluorescence	Thermo 62,249
Secondary antibody	Anti‐Mouse IgG DyLight 594	Red fluorescence	Vector DK‐2594
Secondary antibody	Anti‐Rabbit IgG DyLight 594	Red fluorescence	Vector DK‐1594

## 3 Statistics

Statistical analyses were performed with software (GraphPad Prism version 9, Boston, MA). Data were reported as mean ± standard deviation (SD). Comparisons were made with Student's *t*‐test for datasets with two groups and one‐way analysis of variance (ANOVA) with Tukey's multiple comparison test for data sets with more than two groups. Statistical significance was defined by *p* < 0.05.

## 4 Results

### 4.1 EV Distribution in Vascular Beds

**FIGURE 1:**
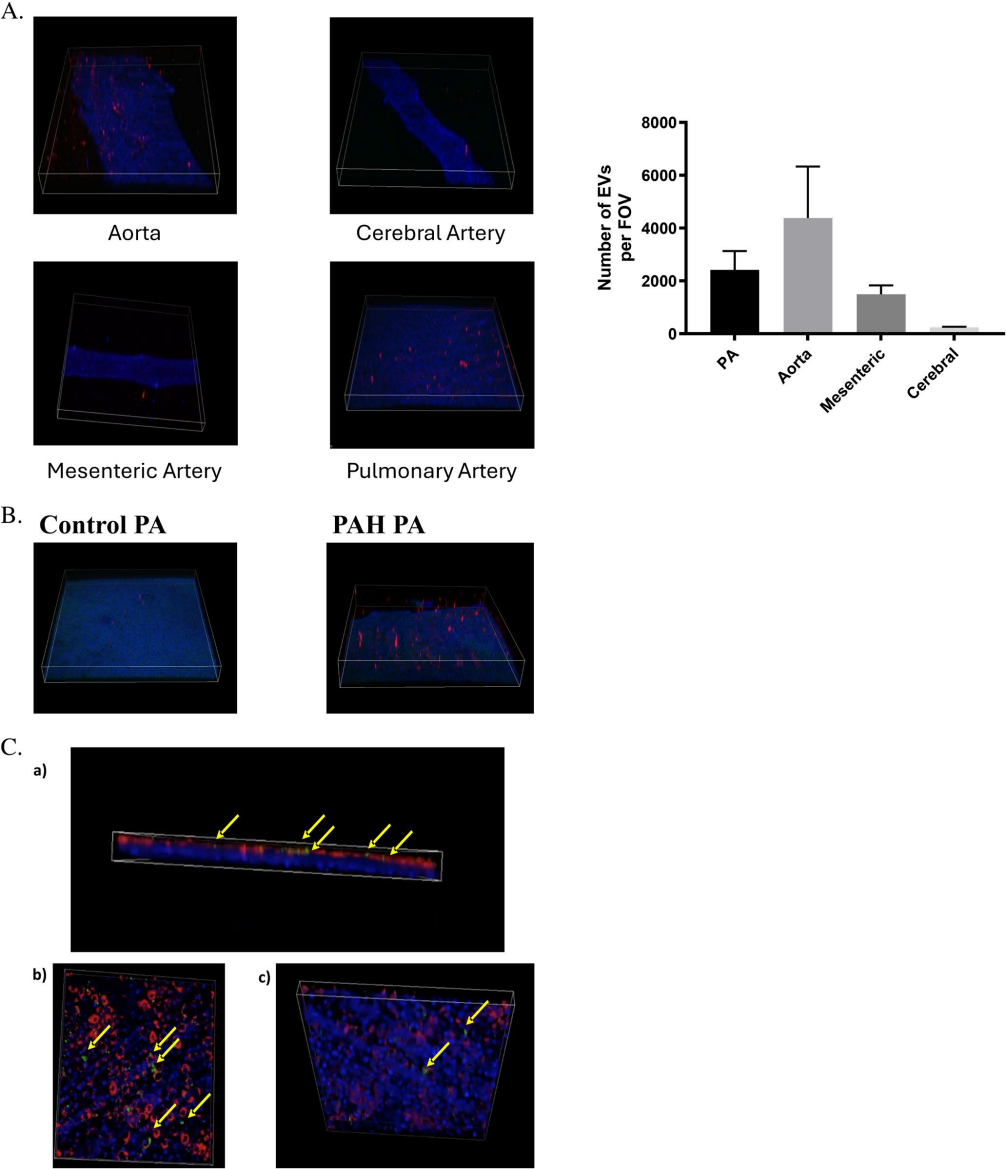
EV uptake and distribution in vivo. (A) Fluorescently labeled EVs (red) in pulmonary arteries of normoxic control versus PAH rats showing increased EVs in PAH (*n* = 6 each). (B) PKH‐labeled EVs (red) injected via tail vein in male Sprague–Dawley rats demonstrate vascular engulfment under physiologic conditions. Nuclei (blue), autofluorescence in matrix/smooth muscle (green). Arrows indicate EVs; mesenteric artery EV is out of plane. *n* = 3. (C) Confocal images of EV uptake in endothelial/PASMC co‐culture on transmembrane. (a) Side view showing endothelial cells (red) and PASMCs (blue). (b) Top view of labeled endothelium. (c) Bottom view of smooth muscle. Yellow arrows indicate EVs.

Confocal microscopy of NucBlue‐stained isolated vessels revealed preferential EV uptake by pulmonary arteries and aorta, with no detection in mesenteric or cerebral arteries (Figure 1A). Pulmonary arteries from PAH rats demonstrated significantly enhanced EV uptake compared to controls, indicating preferential targeting of injured vasculature (Figure 1B). To identify the cellular target within vascular beds, we employed a transwell co‐culture system with PKH26‐labeled PECs on the upper surface and PASMCs on the inverted surface, mimicking vascular architecture [9]. PKH67‐labeled EVs were applied to the endothelial compartment and monitored via confocal microscopy. No labeled EVs were observed translocating to the PASMC layer, demonstrating that endothelial cells are exclusively responsible for endothelial‐derived EV uptake (Figure 1C). These findings establish that pulmonary endothelium serves as the primary site for EV incorporation, with enhanced uptake in diseased vessels, while smooth muscle cells remain uninvolved in this process despite their proximity and importance in vascular crosstalk.

### 4.2 EVs Are Engulfed by Endothelial Cells and Colocalization With Intracellular Organelles

**FIGURE 2:**
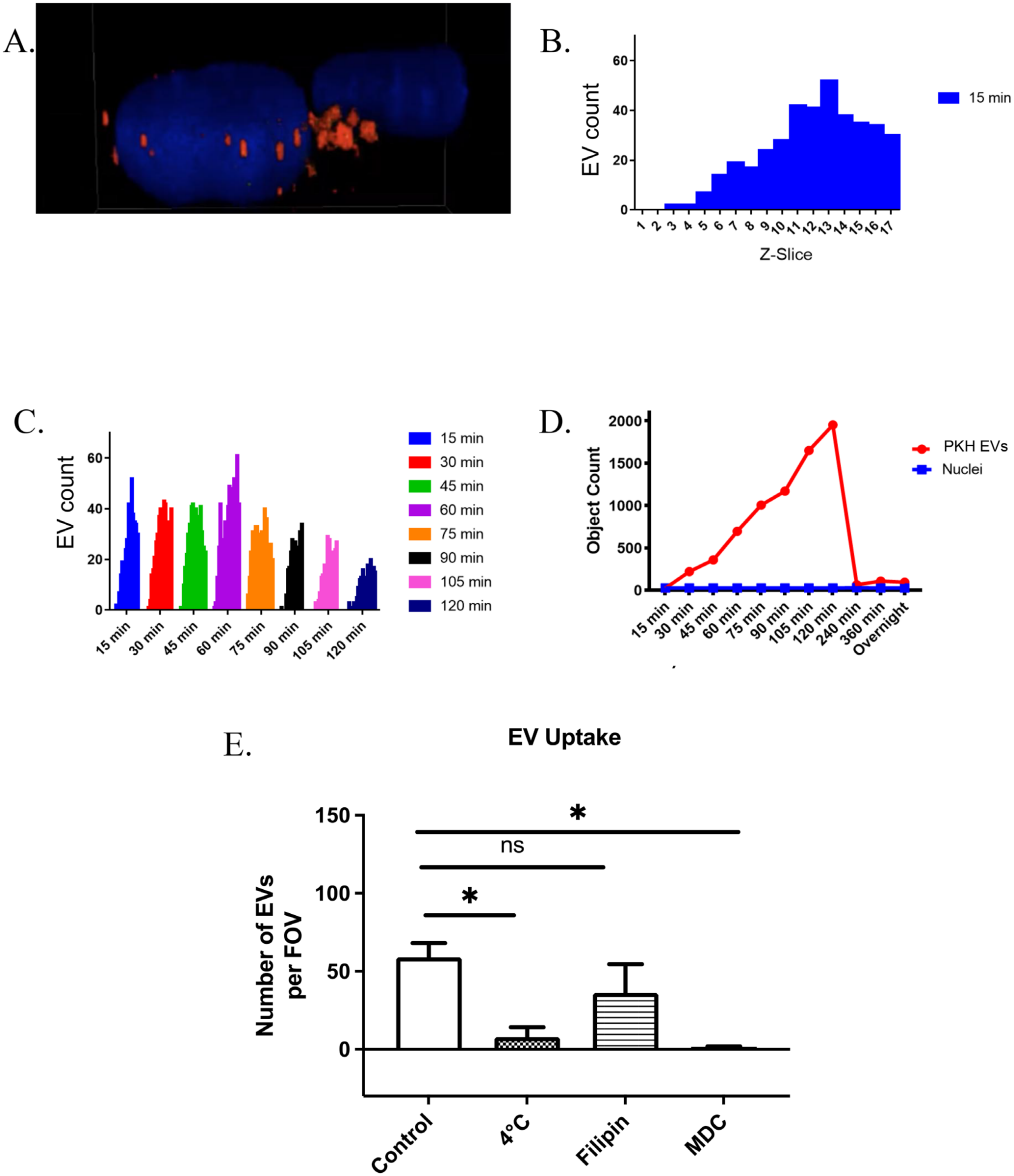
EVs are engulfed by endothelial cells. (A) Confocal *z*‐stack side view of PKH26‐labeled EVs (red) applied to endothelial cells for 1 h. Nuclei (blue) show EVs are internalized. (B) Representative Z‐slice analysis at 15 min time‐point. (C) Cumulative data from (B). (D) Overnight analysis showing minimal intact EV detection after ~4 h. (E) EV uptake is energy‐dependent (4°C inhibition) and clathrin‐mediated (MDC inhibition). Filipin pretreatment (59 ± 9.09 vs. 36 ± 18.5), 4°C (7.857 ± 6.375), or MDC pretreatment (1.5 ± 0.4014), *n* = 5–10. *p < 0.05.

Confocal imaging demonstrated preferential EV accumulation in pulmonary vascular beds compared to cerebral or mesenteric vasculature. Co‐culture experiments revealed that endothelial cells, not pulmonary smooth muscle cells, were responsible for EV uptake. To determine whether endothelial EVs undergo intact engulfment or membrane fusion with recipient endothelium, we performed overnight time‐course imaging with *Z*‐stack analysis (Figure 2A). Our data showed significant engulfment of intact EVs with no evidence of membrane intercalation (Figure 2B). EVs accumulated intracellularly near the nucleus rather than spreading on cell surfaces, indicating that membrane fusion is unlikely to be the primary uptake mechanism for endothelial EV‐to‐endothelial cell transfer (Figure 2C). *Z*‐stack analysis across multiple fields confirmed the complete absence of detectable intact EVs following overnight incubation, suggesting intracellular processing (Figure 2D).

To characterize the endocytosis mechanism, we investigated energy dependence and specific uptake pathways. EV internalization was completely inhibited at 4°C, confirming energy‐dependent uptake. We distinguished between clathrin‐ and caveolin‐mediated endocytosis using monodansylcadaverine (MDC) and filipin as specific inhibitors, respectively. MDC treatment completely inhibited EV uptake, whereas filipin had a minimal effect (Figure 2E). These findings establish that endothelial EV internalization occurs through energy‐dependent, clathrin‐mediated endocytosis.

To understand EV cargo delivery, we examined organelle colocalization. Following EV treatment, cells were fixed and immunostained for specific organelles (Figure S1A,B). Hyperspectral imaging and CellProfiler analysis revealed significant colocalization with early endosomes, trans‐Golgi, and lysosomes. Early endosome localization confirms expected endocytic trafficking (Figure S1C).

## 5 Discussion

EVs play crucial roles in injury and homeostasis. While EV release mechanisms, contents, and membrane structure are well‐studied, uptake and intracellular processing remain poorly understood [10]. Pulmonary arteries from PAH rats demonstrated significantly enhanced EV uptake compared to controls, indicating preferential targeting of injured vasculature. This increased uptake in diseased pulmonary arteries suggests that vascular injury or remodeling in PAH creates a microenvironment that is more receptive to EV incorporation, potentially due to altered endothelial surface markers or increased permeability. It is possible that the pathological state of the pulmonary endothelium, characterized by inflammation, cellular activation, or disrupted barrier function, facilitates greater interaction and internalization of circulating EVs. These findings underscore the potential for EVs to selectively target and deliver cargo to sites of vascular injury, which may have important implications for therapeutic strategies aimed at modulating endothelial function in pulmonary arterial hypertension. Our findings demonstrate preferential uptake by pulmonary endothelium, confirmed through co‐culture models showing exclusive endothelial localization of intact EVs, indicating that EV processing occurs within the endothelial monolayer.

To identify the cellular target within vascular beds, we employed a transwell co‐culture system with PKH26‐labeled PECs on the upper surface and PASMCs on the inverted surface, mimicking vascular architecture [8]. PKH67‐labeled EVs were applied to the endothelial compartment and monitored via confocal microscopy. No labeled EVs were observed translocating to the PASMC layer, demonstrating that endothelial cells are exclusively responsible for endothelial‐derived EV uptake. These findings establish that pulmonary endothelium serves as the primary site for EV incorporation, with enhanced uptake in diseased vessels, while smooth muscle cells remain uninvolved in this process despite their proximity and importance in vascular crosstalk.

EV uptake requires energy and occurs via clathrin‐dependent endocytosis [10]. Uptake frequency increased over 2 h, followed by significant detection loss, suggesting either uptake saturation or rapid intracellular processing rendering EV membranes undetectable.

We investigated EV colocalization with intracellular organelles to understand content release and membrane distribution mechanisms. Within 1 h, significant colocalization occurred not only with early endosomes, as expected due to clathrin‐dependent endocytosis, but also with trans‐Golgi and lysosomes. These findings suggest EVs possess intracellular localization signals directing them toward lysosomal degradation, membrane recycling, or protein delivery via trans‐Golgi. Future studies will examine EV processing within these organelles to elucidate recycling mechanisms and content delivery pathways.

## 6 Limitation

This study is limited by its use of PKH dyes for EV labeling without dye‐only controls. PKH dyes form aggregates and transfer nonspecifically; the observed intracellular signals may reflect dye artifacts rather than authentic EVs.

## Author Contributions

**Aritra Bhadra:** conceptualization, investigation, formal analysis, writing – original draft preparation, reviewing, and editing. **April Scruggs:** methodology, visualization, investigation. **April Haven:** methodology. **Morgan Malone:** methodology, visualization, investigation. **Natalie Bauer:** conceptualization, formal analysis, writing – original draft preparation, reviewing, and editing.

## Funding

The authors have nothing to report.

## Disclosure

The authors have nothing to report.

## Conflicts of Interest

The authors declare no conflicts of interest.

## Supporting information


**Figure S1:** Supporting Information.

## Data Availability

The data that support the findings of this study are available on request from the corresponding author.
