# Supplementary material for: Pulmonary Endothelial Extracellular Vesicles Preferentially Interact and Are Processed in Pulmonary Endothelial Cells
Source: FASEB Bioadv. 2026 Apr 2;8(4):e70099. doi: 10.1096/fba.2025-00327 (PMC13052107; doi:10.1096/fba.2025-00327)
Supplement: Supplementary file 1 — Figure S1: Supporting Information. [file FBA2-8-e70099-s001.pdf]

Figure S1

Figure S1

A.

cis golgi | trans golgi | Mitochondria | Early Endosome

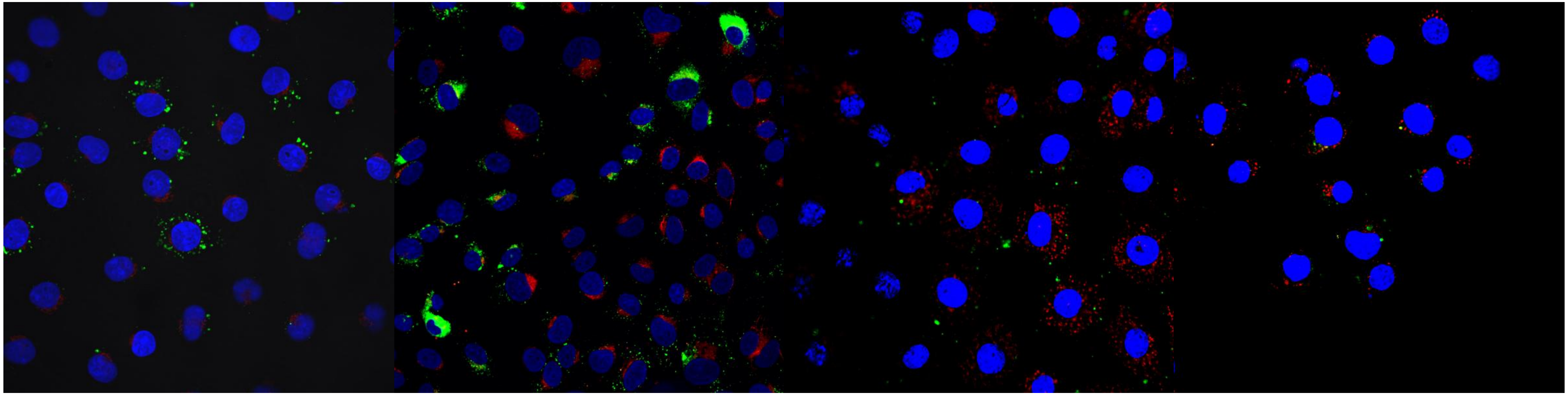

B.

Lysosome

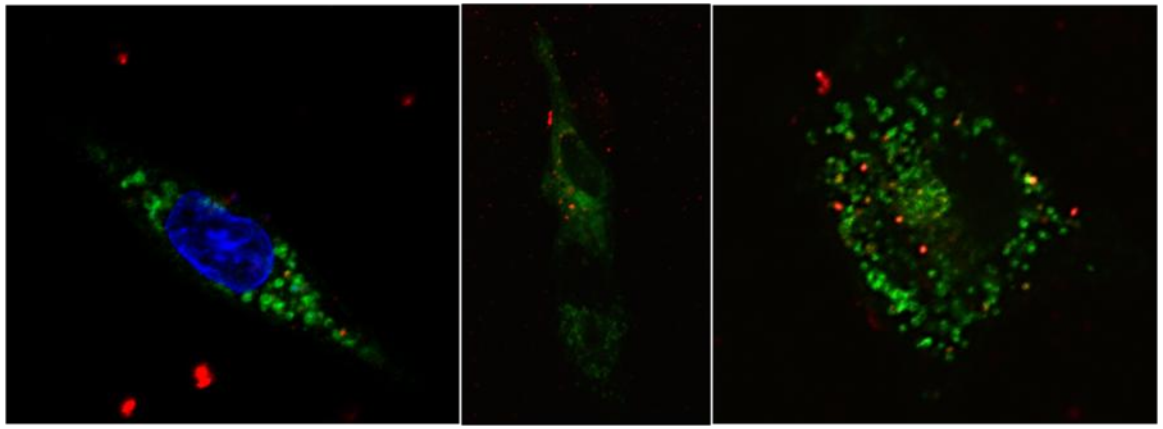

Figure S1

C.

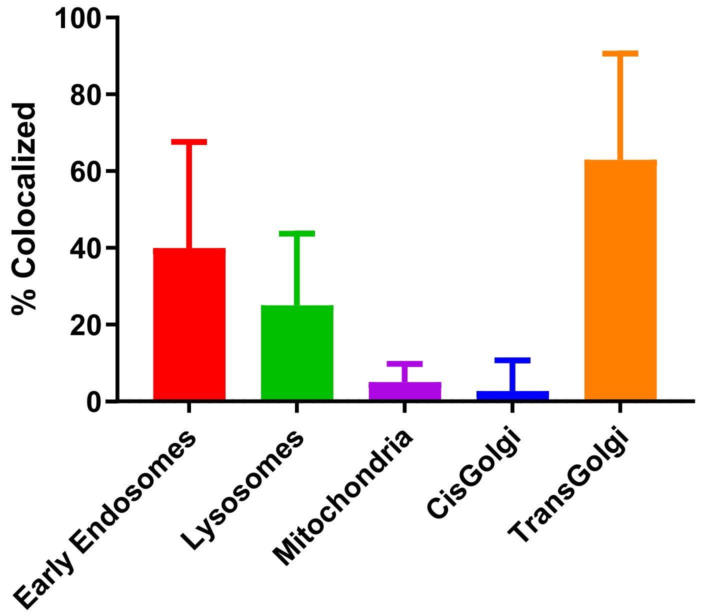

100
80
60
40
20
0
% Colocalized
Early Endosomes
Lysosomes
Mitochondria
CisGolgi
TransGolgi

Figure S1. Co-localization of EVs with intracellular organelles.

A. EVs (PKH67, green) do not colocalize with cis-golgi (GRASP65, red). EVs colocalize with trans-golgi network (TGN38, red). Nuclei (DAPI, blue). EVs do not colocalize with mitochondria (MitoTracker Red) in live cells. Nuclei (Hoechst, blue). EVs colocalize with EEA1+ early endosomes (red).

B. EVs (PKH26, red) show colocalization with lysosomes (LAMP-1, green) in transfected cells.

C. Quantification of EV–organelle colocalization. Colocalization was quantified using CellProfiler across n=5 independent biological replicates, with one field of view (~5–8 cells) imaged per replicate. Percent colocalization was defined as the proportion of EVs exhibiting fluorescence overlap with a given organelle marker relative to total EVs in the field. Each data point represents an individual colocalization event pooled across all five experiments, not an independent replicate; point counts vary by organelle due to differing interaction frequencies.
